# The Influence of Injection Rate on the Hypnotic Effect of Propofol during Anesthesia: A Randomized Trial

**DOI:** 10.1371/journal.pctr.0010017

**Published:** 2006-07-28

**Authors:** Jasmin Blum, Eberhard Kochs, Nicole Forster, Gerhard Schneider

**Affiliations:** Department of Anesthesiology, Technische Universität München, Klinikum rechts der Isar, Munich, Germany

## Abstract

**Objective:**

Previous studies suggested that slow injection of propofol may increase the hypnotic effect during induction of anesthesia. The aim of the present study was therefore to investigate whether injection rate of propofol has an influence on its maximum effect.

**Design:**

Randomized, single-blind trial.

**Setting:**

This study has been carried out in the operating rooms of a university hospital. An anesthesiologist and a resident performed the study with the aid of changing nursing staff.

**Participants:**

We investigated 99 unpremedicated patients aged 18 to 60 years with American Society of Anesthesiologists (ASA) physical status 1–3.

**Interventions:**

Anesthesia was induced by intravenous injection of propofol (2 mg/kg). Propofol was manually injected in group 1 over a period of 5 s; in group 2 (120-s injection interval), and in group 3 (240-s injection interval), propofol was administered by an injection pump. After loss of consciousness, mask ventilation was performed with 100% oxygen. Bispectral index (BIS) was used to measure the hypnotic effect of propofol. After the decrease of BIS to the minimum value (i.e., maximum hypnotic effect) and the following increase of BIS to 60, the study period was finished and anesthesia was performed according to clinical criteria.

**Outcome Measures:**

We analyzed whether injection speed has an influence on the maximum hypnotic effect of a given dose of propofol (2 mg/kg).

**Results:**

BIS_min_ marks the maximum electroencephalogram (EEG) effect of the propofol bolus as measured by the BIS. The lowest mean BIS_min_ was measured in group 1 (28.7 ± 10.3). In group 2, BIS_min_ was 33.0 (±13.9), and in group 3, BIS_min_ was 36.4 (±11.0). There were no significant differences between group 2 and groups 1 or 3, but there were significant differences between groups 1 and 3. In group 1, BIS_min_ was reached after 102.91 s (±44.20), in group 2 after 172.33 s (±29.76), and in group 3 after 274.21 s (±45.40). These differences were statistically significant for all comparisons. In summary, the lowest value for BIS_min_ was achieved in the group with the fastest rate of propofol injection (group1, 5 s). The highest BIS_min_ was obtained in the group with the slowest rate of injection (group 3, 240 s). The hemodynamic parameters were not significantly different among groups.

**Conclusions:**

The hypnotic peak effect of propofol is lower with extremely slow injection (240 s versus 5 s). For clinically usual injection rates (5 s and 120 s), there was no significant difference in propofol peak effect.

## Editorial Commentary

**Background:** Propofol is an injectable compound that is commonly used to bring about anesthesia in adults and in children aged more than three years. The rate at which propofol is injected is thought to affect the total dose of the drug that's needed to achieve loss of consciousness and lowered blood pressure during anesthesia. Previous trials have looked at the effect of different injection rates on anesthesia (time taken to lose consciousness, and degree of consciousness). In this trial of 99 patients scheduled for elective surgery, the researchers studied the effect of three different propofol injection rates. Patients were randomized to receive propofol injected over 5 s, 120 s, or 240 s. In each group the total dose of propofol (per kilogram of a patient's bodyweight) was the same. The main measure used to assess anesthetic effect was the bispectral index. This is a method of translating information from an electroencephalogram (graph showing electrical activity in the brain) into a standard measurement that reflects the patient's level of consciousness. The researchers also recorded time to loss of consciousness, i.e., when patients stopped responding to commands, and took blood pressure measurements.

**What this trial shows:** The researchers found that anesthetic effect, as measured using the bispectral index, was greatest in the patients who had received the fastest injections as compared with those who had received slower injections. However, the difference was only significant when comparing the fastest injection (5 seconds) with the slowest (240 seconds). In addition, the time taken to achieve anesthesia (as measured using the bispectral index), and time to loss of consciousness (as indicated by no response to commands), were lowest in patients who had the fastest injections; these differences were also significant. The researchers did not find an effect of the different injection rates on maximum and minimum blood pressure during the trial.

**Strengths and limitations:** The trial recruited enough patients to properly assess whether patients receiving different injection rates would have different responses to anesthesia. A limitation, acknowledged by the authors, is that the bispectral index uses a commercial computer program to interpret electroencephalograms and to produce a number value for anesthetic effect. Some evidence suggests that the output of the computer program may not correlate precisely with level of consciousness, and as the algorithm is not public, any irregularities in the way it works cannot be discovered by researchers outside the company. It is also of note that the slowest injection rate used by the researchers, 240 seconds, is not normally used in clinical practice.

**Contribution to the evidence:** The results of this study support those from a few other small randomized trials that faster injections of propofol achieve a larger anesthetic effect, and more quickly. However, the effect of injection rate on blood pressure is less clear; this study does not show any differences in the effect of injection rate on blood pressure, but other randomized trials have found an association.

## INTRODUCTION

The clinical daily routine indicates that slowing the rate of administration of propofol can lead to a reduction of up to 50% in the dose of propofol required to achieve the onset of a clinical endpoint of anesthesia (i.e., loss of consciousness [LOC]) when titrating to effect. Therefore, it has been concluded that a slow injection requires a smaller dose of propofol as the graded effect is weakened by fast injection [1,2]. This conclusion contradicts the pharmacologic consideration that a fast injection would lead to a higher peak concentration, and, in consequence, to a higher peak effect in the brain.

The present study was designed to measure the electroencephalogram (EEG) peak effect of a propofol bolus (2 mg/kg) injected with different infusion rates.

Although it is known that propofol has cardiovascular effects, the influence of injection rate on these cardiovascular changes is less clear. Gillies and Lees [3] found that faster injection rates of propofol caused greater reductions in blood pressure (BP). Other similar studies did not show differences in BP for different injection rates [4]. An additional aim of this study was therefore to investigate the influence of different injection rates on hemodynamic parameters.

## METHODS

### Participants

We investigated 99 patients, of both sexes, from 18 to 60 years old with American Society of Anesthesiologists (ASA) physical status 1–3. All patients were scheduled for elective surgery under general anesthesia. Exclusion criteria were emergency surgery, obesity (Broca index > 25%), indication for rapid sequence induction, administration of drugs that affect the central nervous system, a history of alcohol or drug abuse, neurological or psychiatric diseases, or contraindications against the use of propofol. The study was carried out in the anesthesia induction rooms of the operating theatre. An anesthesiologist and a resident performed the study with the aid of changing nursing staff.

### Interventions

Having approval from the university's ethics committee, and after written informed consent was obtained, this prospective, single-blind study was performed for 99 patients. No premedication was given prior to induction.

Baseline heart rate (HR) and BP were measured within 72 h before surgery and immediately before induction of anesthesia. An intravenous catheter was inserted into the brachial vein and infusion of lactated Ringer's solution was started. Anesthesia was induced by intravenous injection of the propofol bolus (2 mg/kg). In group 1, propofol (propofol Abbott 1%) was manually injected over a period of 5 s; in group 2 (120-s injection interval) and group 3 (240-s injection interval), propofol was administered by an injection pump. When spontaneous respiration ceased, patients were ventilated by face mask with 100% O_2_. The following clinical information was recorded: LOC and loss of lash reflex (LOL). LOC was defined as the time when the patient stopped responding to commands (“squeeze my hand”). After the maximum hypnotic effect of propofol, a decrease of the hypnotic component of anesthesia was indicated by an increase of BIS. As soon as BIS was increased to index values of 55–60, the investigation was completed and additional propofol, opioid, and muscle relaxant were given. The patients' tracheas were intubated and anesthesia was continued according to standard clinical practice.

Patients were attached to a 3-lead electrocardiogram, pulse oxymeter, and BP cuff (Datex AS/3 Compact Monitor). Additionally, a two-channel fronto-temporal EEG (Aspect A-1000, Aspect Medical Systems, Newton, Massachusetts, United States) was measured. EEG electrodes were positioned according to the manufacturer's recommendations at AT1, AT2, Fpz (common reference), F1 (ground). The high pass was set to 0.5 Hz; no low pass was used. After attaching the electrocardiogram, BP cuff, and pulse oxymeter, EEG electrodes were attached and impedance was checked and maintained below 1.5 kΩ. Digital EEG and calculated EEG parameters were recorded continuously. BP and HR were measured every minute. Data were stored on a PC (Datalogger Software, Aspect Medical Systems).

### Objectives

The primary goal of the study was to investigate whether faster injection rates of propofol lead to an increased maximum effect as measured by BIS. Secondary criteria were differences in onset times and differences in hemodynamic parameters.

### Outcomes

The main parameter investigated in this study was the maximum hypnotic effect as indicated by the minimum BIS value (BIS_min_). In addition, the times until LOC (t-LOC), LOL (t-LOL), and BIS_min_ (t-BIS_min_) were measured. Furthermore, we analyzed BIS at LOC (BIS-LOC), BIS at LOL (BIS-LOL), and BIS 30 s after LOC (BIS-LOC^+30s^). The minimum and maximum differences to baseline in HR (HR_min_, HR_max_), mean arterial pressure (MAP_min_, MAP_max_), and systolic and diastolic BP (BP^SYS^_min_, BP^DIA^_min_, BP^SYS^_max_, BP^DIA^_max_) were calculated.

### Sample Size

With a sample size between 13 and 33 patients per group, a one-factor ANOVA reaches 80% power and a significance level of 5% to detect a difference in mean values which is characterized by a variance of mean values, V = Σ(μ−μ)^2^/3, in the range of 20.5 to 55.5. A standard deviation of 14.00 is the basis of this calculation. The ranges described were derived by simulations on the basis of BIS data measured during induction of anesthesia (mean BIS 36, standard deviation 14). Minimum requirement was the detection of a difference of mean values in the range of one standard deviation (14.00). As a consequence of the imprecision of the underlying assumptions, a blind interim analysis was performed after *n* = 20 patients per group. Results of this analysis were not made available to anybody involved in the clinical study. On the basis of this analysis, the sample size was corrected to the maximum of 33 per group.

### Randomization—Sequence Generation

Blocked randomization was performed. The randomization list was generated as follows: the first block consisted of 60 patients (three groups with 20 patients, reflecting three different injection rates), allowing a blind interim analysis at this point. The second block consisted of an additional 39 patients, 13 in each of the three groups. For each block, a Microsoft Excel table was generated with the corresponding groups in column 1. In column 2, a number was added using the “random number” function of Microsoft Excel. Next, the tables were sorted by values in column 2 (in ascending order), which rearranged the group assignments according to the randomly generated numbers.

### Randomization—Implementation

According to the computer-generated list, envelopes with group assignments were sealed and arranged in the order of the randomization list. This order was maintained during patient enrollment.

### Randomization—Allocation Concealment

After written informed consent had been obtained, the patients were randomly assigned to one of the three different injection rates as the responsible anesthesiologist opened the next envelope.

### Blinding

Only patients were blind to the different injection rates.

### Statistical Methods

Blind interim analysis of BIS_min_ values did not reveal significant differences between groups. For all 99 patients, data analysis was performed. ANOVA and subsequent post hoc tests with Bonferroni correction (*p* < 0.05) were performed to identify differences in BIS_min_ values between groups. In an exploratory approach, times from injection start to LOC and BIS_min_ also were analyzed. A Kruskal-Wallis test and ANOVA were performed to detect differences in demographic values between groups. Data are presented as mean ± SD.

## RESULTS

### Participant Flow

In the present study, all patients who were asked agreed to take part in the trial. We assume that the reason for this high enrollment rate is the fact that consent to the study did not result in a major deviation from standard clinical practice and did not cause additional risk for the patients. In fact, the additional EEG monitoring may have added safety for the patients.

**Figure 1:**
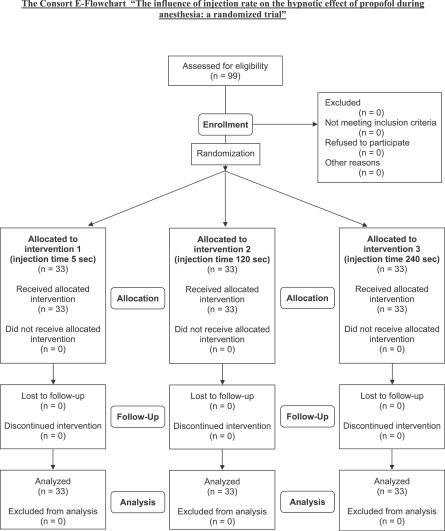
The CONSORT Flowchart Illustrates Patient Enrollment, Allocation, Follow-Up, and Analysis

All patients received the treatment as allocated, no patient was excluded, and no deviations from the study protocol occurred (see Figure 1, the CONSORT flowchart).

### Recruitment

The patients were recruited from March to December 2003. All the patients were selected and included in the study within 72 h before surgery.

### Baseline Data

Table 1 shows demographic data of the patients. There were no significant differences among the three groups (Table 1).

**Table 1:**
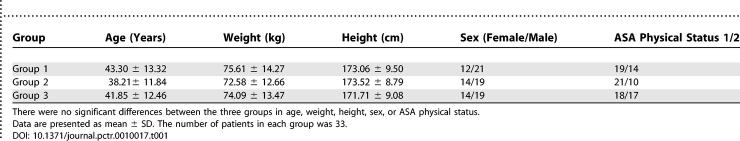
Demographic Data of the Patients (*n* = 99)

### Numbers Analyzed

All 99 patients who underwent random allocation were analyzed according to group assignment, no patient was excluded from the analysis.

### Outcomes and Estimation 

#### Maximum effect: BIS_min_.

**Figure 2:**
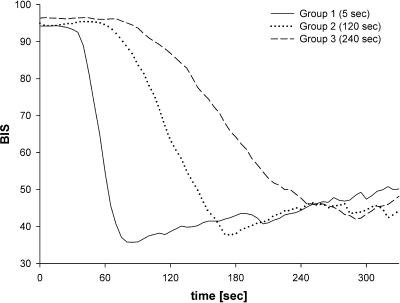
Mean BIS Developing for Each of the Three Groups The figure shows the sharp BIS decrease and the fast reaching of BIS_min_ in group 1. In contrast to group 1, in group 3 the BIS curve runs very flat and it takes a longer time to reach BIS_min_.

BIS_min_ marks the maximum EEG effect of the propofol bolus as measured by the EEG bispectral index (BIS). ANOVA revealed a statistically significant difference in BIS_min_ between groups (*p* < 0.05). The lowest mean BIS_min_ was measured in group 1 (28.7 ± 10.3). In group 2, BIS_min_ was 33.0 (±13.9), and in group 3, BIS_min_ was 36.4 (±11.0). This difference was statistically significant only between groups 1 and 3, whereas BIS_min_ of group 2 showed no significant difference from either of the other two groups (Table 2). Figure 2 shows the mean BIS curve progression for each of the three groups.

**Table 2:**
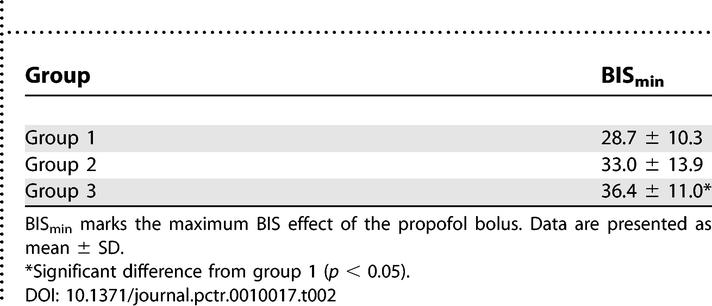
The Maximum BIS Effect (BIS_min_)

#### Time from start of injection to maximum effect BIS_min_.

In group 1, t-BIS_min_ was reached after 102.91 s (±44.20), in group 2 after 172.33 s (±29.76), and in group 3 after 274.21 s (±45.40). These differences were statistically significant for all comparisons (Table 3).

**Table 3:**
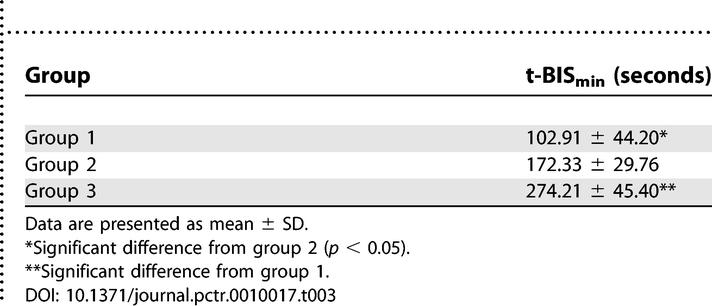
t-BIS_min_

#### Time from end of injection to maximum effect—adjusted t-BIS_min_.

As a consequence of the decreasing bolus rate, there is an increasing time delay before the total amount of propofol is given. Therefore, t-BIS_min_* was calculated: the duration of injection (5 s, 120 s, 240 s) was subtracted from t-BIS_min_. By this approach, the time to BIS_min_ after injection of the total dose of propofol was calculated. t-BIS_min_* was 99.85 s (±47.87) in group 1, 52.41 s (±30.23) in group 2, and 32.69 s (±43.77) in group 3.

#### t-LOC and t-LOL.

t-LOC was defined as the time from the beginning of the propofol injection to LOC. t-LOC in group 1 was 35.76 s (±20.62), in group 2 108.18 s (±17.98), and in group 3 177.73 s (±43.82). t-LOC increased in every group by approximately 70 s (*p* < 0.05).

t-LOL was defined as the time from the beginning of propofol injection to loss of eyelash reflex. t-LOL is clinically used as a sign of deeper anesthesia and occurs after t-LOC. t-LOL was 49.24 s (±12.63) in group 1, in group 2 134.24 s (±20.31), and 210.45 s (± 44.95) in group 3 (*p* < 0.05).

#### BIS-values at LOC and LOL.

BIS-LOC in group 1 was 91.3 (±8.0), in group 2 75.7 (±10.4), and in group 3 66.8 (±14.1). BIS at the time of LOL was 74.8 (±25.1) in group 1, 55.7 (±19.0) in group 2, and 51.8 (±15.2) in group 3. For both, BIS-LOC and BIS-LOL values in group 1 (5 s) were significantly higher than in group 2 (120 s) or group 3 (240 s). There were no significant differences between group 2 and group 3.

#### BIS-values 30 seconds after LOC.

BIS-LOC^+30s^ is the BIS value 30 seconds after LOC. BIS-LOC^+30s^ was 49.1 (±25.2) in group 1, 49.9 (±19.9) in group 2, and 50.7 (±14.6) in group 3. There were no significant differences in BIS-LOC^+30s^ between the three groups.

#### Hemodynamic parameters.

Baseline hemodynamic values (HR, MAP, BP^SYS^, and BP^DIA^) were calculated from the mean values measured 72 h before surgery and before induction of anesthesia. Baseline hemodynamic measurements did not show significant differences between the groups (Table 4).

**Table 4:**
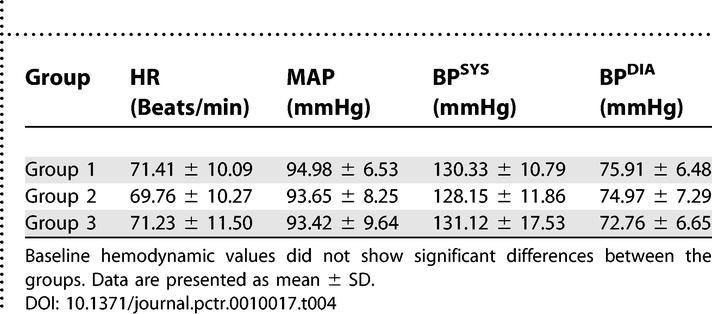
Baseline Hemodynamic Values

The maximum and minimum values of hemodynamic parameters from the beginning of propofol injection to the end of the investigation were identified. These minima and maxima did not show significant differences between groups (Tables 5 and 6).

**Table 5:**
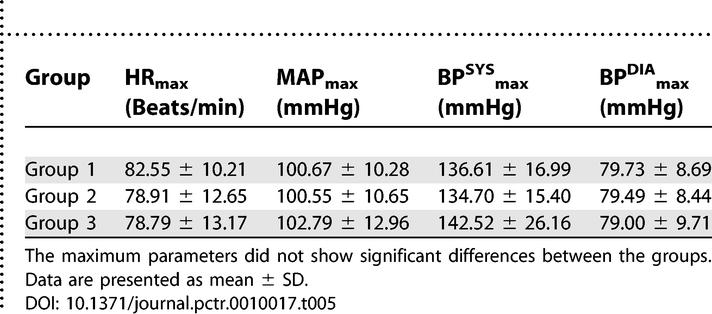
The Maximum Values of Hemodynamic Parameters

**Table 6:**
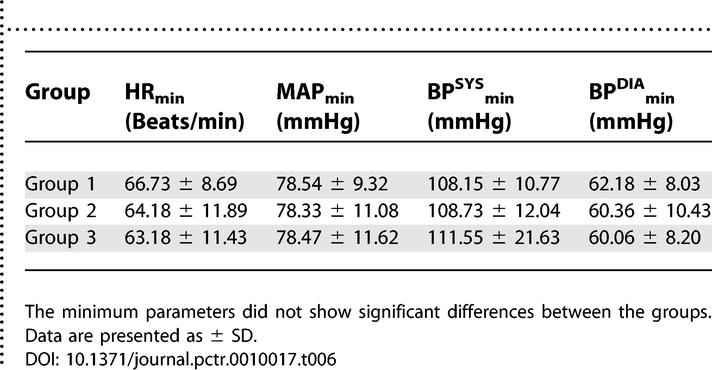
The Minimum Values of Hemodynamic Parameters

### Adverse Events

During induction of anesthesia, surgery, and at the recovery room no side effects were observed, neither in groups 1 and 2, nor in group 3.

## DISCUSSION

### Interpretation

The study shows an influence of propofol injection rate on its maximum effect as measured by EEG BIS. After extremely slow induction of anesthesia (240 s), the propofol peak effect is significantly lower than after rapid injection (5 s), as indicated by higher BIS values. The EEG was used to determine the effect of different rates of propofol infusion. A possible limitation of the study design is the use of BIS as an endpoint. The correlation between propofol concentrations and BIS values may not be entirely linear. In particular, increasing concentrations of anesthetics may be misinterpreted as a lighter level of hypnosis [5]. During propofol anesthesia, this phenomenon was observed at the onset of burst suppression [6]. It is known that in the range of 20–30, a “plateau” in the BIS algorithm exists. The BIS will only decrease if a burst suppression ratio higher than 40 appears. As the BIS algorithm is proprietary, one can only speculate about the reasons for this nonlinearity. The use of a proprietary algorithm induces additional (unknown) sources of error. Therefore, the use of proprietary “depth of anesthesia” indices has recently been criticized, and it has been suggested that such monitors not be used until the algorithms have been revealed [7]. In the current study, however, we accepted the limitation. In particular, it has been shown that BIS correlates with propofol target concentrations [8,9]. Therefore, we decided to use BIS as a measure of propofol peak effect despite its known limitations. As indicated by differences in BIS_min_, rapid injection (5 s) of propofol has a higher peak effect than very slow injection (240 s). The sample size of the study was designed to detect a difference of 15 or more in BIS values. Therefore, a smaller difference between 5-s and 120-s or between 120-s and 240-s injection rates can not be excluded.

The faster the injection rate, the faster specific effects (LOC, LOL, maximum peak effect) were reached. As the analysis of t-BIS_min_* and the time from end of injection to BIS_min_ shows, the injection rate is an intrinsic part of these results.

### Generalizability

In the present study, hemodynamic parameters were stable in all groups. This is consistent with previous studies mentioned above [1,2]. In two groups of patients (18–50 y/60 y, ASA physical status 1–2), the effect of different injection rates (25 mg/min, 50 mg/min, 100 mg/min, 200 mg/min, bolus) on propofol effect were studied. There were no significant differences in HR, BP^SYS^, or BP^DIA^ [2]. A study in younger patients (18–55 y, injection rates 50 mg/min, 100 mg/min, 200 mg/min) also did not show significant changes in BP [1]. In contrast, a study in elderly patients (>60 y, ASA physical status 1–4) found significantly less decrease in BP with slow injection of propofol [10]. In contrast to this study, and similar to the above-mentioned studies, elderly patients and patients with preexisting diseases were not included in our study. This, and the volume preload of lactated Ringer's solution, may explain why none of our patients showed hemodynamic instability.

The greater effect of quickly injected propofol is consistent with pharmacokinetic principles: fast injection rate increases peak concentration, which will subsequently lead to an increased drug peak effect. This is supported by studies that indicate an increased effect site concentration after fast injection. In an animal study with sheep, catheters were inserted into the carotid artery, sinus sagittalis, and the right atrium. A propofol bolus (100 mg) was administered with different injection rates (200 mg/min, 50 mg/min, 20 mg/min). The peak concentration of propofol was found to increase with faster injection [11]. In concordance with these findings, results of the present study indicate a decreased propofol peak effect with slow injection of propofol.

### Overall Evidence

Previous studies showed that the duration of injection has an influence on the total dose of propofol that is necessary for LOC. In 1992, Peacock et al. showed that rapid injection of propofol (200 mg/min) leads to significantly faster LOC when compared with a slow injection (25 mg/min) [2]. Even more interesting, the propofol dose required to induce LOC was significantly lower in patients who received the slow injection. Results of this study were consistent with previous studies in adult [1] and elderly patients [10]. Therefore, the authors concluded that the necessary dose of propofol for induction of anesthesia is lower when the duration of injection is longer. This seems to contradict our results and the described pharmacokinetic principles. This difference can be explained by the different endpoints used in the clinical studies. LOC, as used in previous studies, is an all-or-none phenomenon that reflects a threshold at the wider scale of hypnotic effects. As a consequence, an “overshoot” effect, i.e., a “deeper” hypnotic level, will not be detected when LOC is used as the endpoint. If propofol is injected until LOC, different doses may result from different injection rates. This is due to pharmacokinetic and pharmacodynamic properties of the drug. After injection, propofol is distributed in the plasma and is transferred to the effect site, i.e., the brain. The propofol fraction in the plasma is referred to as “drug in transit.” In previous studies, a constant rate of propofol was given until LOC occurred. LOC, however, reflects the effect of propofol at the effect site, i.e., in the brain, whereas the total dose of propofol given includes the propofol “in transit,” i.e., in the plasma. After termination of propofol injection at LOC, the total amount of propofol that has been injected is transferred to the brain, and subsequently the hypnotic level will increase after LOC (overshoot reaction). With a constant transit time to the effect site, faster injection rates will lead to higher doses of propofol in transit, i.e., a higher total dose of propofol. This may explain why propofol injection until LOC as an endpoint will result in propofol doses that increase with increasing injection rates. In summary, a slow injection of propofol leads to improved titration when administered to clinical effect, whereas the peak effect of a given dose seems higher after rapid injection.

## SUPPORTING INFORMATION

CONSORT Checklist Click here for additional data file. (48 KB DOC)

Trial Protocol Click here for additional data file. (57 KB DOC)

## Abbreviations

ASA: American Society of Anesthesiologists
BIS: bispectral index
BIS_min_: minimum BIS
BP: blood pressure
BP_SYS_: systolic blood pressure
BP_DIA_: diastolic blood pressure
EEG: electroencephalogram
h: hours
HR: heart rate
LOC: loss of consciousness
LOL: loss of lash reflex
MAP: mean arterial pressure
s: seconds
t-BIS_min_*: t-BIS_min_ adjusted
