# Supplementary material for: The Influence of Injection Rate on the Hypnotic Effect of Propofol during Anesthesia: A Randomized Trial
Source: PLoS Clin Trials. 2006 Jul 28;1(3):e17. doi: 10.1371/journal.pctr.0010017 (PMC1523225; doi:10.1371/journal.pctr.0010017)
Supplement: Trial Protocol [file pctr.0010017.sd002.doc]

# KLINIK FÜR ANAESTHESIOLOGIE

**KLINIKUM RECHTS DER ISAR**

**TECHNISCHE UNIVERSITÄT MÜNCHEN**

(Direktor: Univ.-Prof. Dr. med. E. Kochs)

Der Einfluß unterschiedlicher Injektionsgeschwindigkeiten auf den EEG-Effekt von Propofol

G. Schneider

Korrespondenzadresse:

Dr. Gerhard Schneider

Klinik für Anaesthesiologie

Klinikum rechts der Isar

Technische Universität München

Ismaningerstr. 22

81675 München

Tel. 089 4140 4291

Fax 089 4140 4886

e-mail: Gerhard.Schneider@LRZ.tum.de

Zusammenfassung

In klinischen Studien ließ sich beobachten, daß bei langsamer Injektion des Einleitungshypnotikums Propofol der Bewußtseinsverlust bei geringeren Gesamtdosen des Medikaments eintritt. Bei Messung der Propofol-Konzentrationen im Gehirn nach einer definierten Dosis Propofol ließ sich jedoch kein Korrelat dieses Befundes nachweisen.

Diese Differenz läßt sich unter Umständen durch Unterschiede im Studienprotokoll erklären, da in ersterer Studie das Medikament als Dauerinfusion mit variabler Gesamtdosis verabreicht wurde. In zweiterer Untersuchung wurde hingegen eine definierte Menge mit unterschiedlicher Geschwindigkeit verabreicht, was dem Vorgehen im klinischen Alltag eher entspricht. Des weiteren wurde in ersterer Studie ein klinischer "Alles-oder-Nichts"-Parameter (Bewußtseinsverlust) verwendet, der keine weitere Differenzierung des Zustands "Narkose" ermöglicht.

Mit vorliegender Untersuchung soll geklärt werden, welchen Einfluß unterschiedliche Injektionsgeschwindigkeit einer definierten Menge des Hypnotikums Propofol auf EEG (quantifizierender Parameter) und hämodynamische Parameter hat.

Hierzu werden bis zu 60-99 Patienten (je nach Zwischenauswertung, s. Fallzahlabschätzung) untersucht. Unter Anwendung des üblichen Narkosemonitorings sowie nichtinvasiver EEG-Ableitung wird hierzu Patienten zur Narkoseeinleitung Propofol in klinisch üblicher Dosierung (2 mg/kg) verabreicht. Dieser Bolus wird in drei Gruppen über einen unterschiedlichen Zeitraum verabreicht (5 sec., 120 sec, 240 sec.). Der maximale EEG-Effekt wird beobachtet, im Anschluß wird die Narkose in üblichem Vorgehen als Kombinationsnarkose fortgeführt.

Die statistische Analyse vergleicht den maximale EEG-Effekt in den drei Gruppen als Maß des hypnotischen Effektes von Propofol. Als Nebenzielgrößen werden Unterschiede in Anflutungszeit und hämodynamischen Effekte zwischen den einzelnen Gruppen verglichen. Das Vorgehen entspricht im gesamten Studienverlauf klinisch üblicher Praxis und beinhaltet keine zusätzliche Gefährdung der Patienten. Die Injektion von Propofol kann in seltenen Fällen zu schmerzhaften Sensationen führen. Die Inzidenz dieser Injektionsschmerzen ist höher bei schneller Injektion. Nahezu vollständig vermeiden läßt sich der Injektionsschmerz bei Verwendung einer ausreichend großen Vene. Aus diesem Grund wird den Patienten die venöse Kanüle in der Ellenbeuge oder am Oberarm gelegt.

Inhaltsverzeichnis

1. Einleitung

1.1 Narkosetiefenbestimmung mittels EEG

1.2 Injektionsgeschwindigkeit

2. Fragestellung

3. Material und Methode

3.1 Einschlusskriterien

3.2 Ausschlusskriterien

3.3 Studienablauf

3.4 Nebenwirkungen

4. Flußdiagramm

5. Statistik

6. Untersucher

7. Literaturverzeichnis

8. Patientenaufklärung

1. Einleitung

1.1 Narkosetiefenbestimmung mittels EEG

Während seit mehr als hundert Jahren klinische Narkosen durchgeführt werden, ist deren genauer Wirkmechanismus bis heute letztlich ungeklärt. Ebenso ist man zur Bestimmung von Narkosetiefe bis heute auf Hilfsparameter wie Herzfrequenz, Blutdruck und autonome Reaktionen des Patienten angewiesen. Seit Jahren wird versucht, ein direktes Maß der Narkosetiefe zu finden. Vielversprechend ist hierbei die Anwendung des EEG, da mit seiner Hilfe das Zielorgan der Narkose, das Gehirn, in seiner Funktion erfaßt wird.1 Das Roh-EEG ist jedoch schwierig zu interpretieren. Deshalb werden verschiedene mathematische Verfahren angewandt, um das komplexe Muster des EEG in einfach zu beurteilende Parameter umzuwandeln. Kernstück dieser Verfahren ist die Fast-Fourier Transformation. Sie zerlegt die komplexen EEG-Wellen in die einzelnen, sie zusammensetzenden Sinuswellen. Diese werden in unterschiedliche Frequenzbereiche aufgeteilt, und deren relative Anteile an der Gesamtkurve wird berechnet. Daraus ergibt sich das EEG Power-Spektrum. In zahlreichen Untersuchungen ließ sich hiermit die Wirkung verschiedener Anästhetika auf das EEG erfassen.2,3 Diese Phänomene lassen sich zur Durchführung pharmakodynamischer und -kinetischer Studien nutzen. Es hat sich jedoch gezeigt, daß eine Vielzahl der beobachteten Veränderungen spezifisch für das jeweils verwendete Pharmakon sind.

Der Bispektral Index (BIS) ist ein neuer, aus dem EEG errechneter multifaktorieller Parameter, der - neben dieser klassischen EEG-Analyse - durch Bispektralanalyse die Beziehung der einzelnen Sinuswellen zueinander berücksichtigt (Phasenkopplung). Es konnte für eine Vielzahl von Medikamenten gezeigt werden, daß der BIS (eine dimensionslose Zahl zwischen 0 und 100) - unabhängig vom verwendeten Medikament - mit der Hypnosetiefe korreliert. Beim wachen Patienten liegt der BIS zwischen 90 und 100, Nullinien-EEG entspricht einem BIS von 0. Ein BIS zwischen 45 und 60 zeigt adäquate Narkose an.4-6

1.2 Injektionsgeschwindigkeit

In klinischen Studien ließ sich beobachten, daß eine langsame Injektion des Einleitungshypnotikums Propofol zur Folge hat, daß der Bewußtseinsverlust bei geringeren Gesamtdosen des Medikaments eintritt.7 Bei Messung der Propofol-Konzentrationen im Gehirn nach einer definierten Dosis Propofol ließ sich jedoch im Tierversuch kein derartiger Effekt nachweisen.8

2. Fragestellung

Mit vorliegender Untersuchung soll geklärt werden, welchen Einfluß unterschiedliche Injektionsgeschwindigkeit einer definierten Menge des Hypnotikums Propofol bei Narkoseeinleitung auf EEG und hämodynamische Parameter hat.

3. Material und Methode

Die vorliegende Untersuchung wird an bis zu 99 Patienten (s. Fallzahlabschätzung) des chirurgischen Routineprogramms durchgeführt, bei denen eine Operation in Vollnarkose durchgeführt wird.

3.1 Einschlusskriterien:

- Alter: 18-60

- körperlicher Allgemeinzustand ASA 1-3

- geplanter chirurgischer Eingriff in Vollnarkose

3.2 Ausschlusskriterien:

- Körpergewicht > 125% des idealen Körpergewichtes (n. Broca)

- Körpergewicht < 75% des idealen Körpergewichtes (n. Broca)

- Notfalleingriff

- nicht nüchterner Patient

- Indikation zur Ileuseinleitung

- Unverträglichkeit oder Kontraindikation gegen eines der verwendeten Medikamente

- Einnahme zentralnervös wirksamer Substanzen

- neurologische oder psychiatrische Vorerkrankungen

3.3 Studienablauf:

Die an der Studie teilnehmenden Patienten werden randomisiert einer von drei Gruppen zugeteilt. In allen Gruppen kommen klinisch übliche Narkoseverfahren zur Anwendung. Die Patienten erhalten keine Medikamente vor Einleitung der Narkose. Bei Ankunft im OP werden die üblichen Überwachungsgeräte angebracht (EKG, Pulsoxymetrie, nicht invasive Blutdruckmessung). Zusätzlich wird bei allen Patienten nicht invasiv ein Zwei-Kanal EEG abgeleitet. Dazu werden nach Reinigung der Haut Klebeelektroden auf Schläfen und Stirn des Patienten angebracht. Hämodynamik- und EEG-Daten werden kontinuierlich auf Computer aufgezeichnet.

Die Ausgangswerte werden wie üblich bestimmt. Hierzu werden Herzfrequenz und Blutdruckwerte zu zwei unterschiedlichen Meßzeitpunkten erhoben: im Zeitraum von 24h vor Einleitung und unmittelbar vor Einleitung. Aus diesen beiden Werten wird der Mittelwert gebildet. Dieser Mittelwert gilt als Ausgangs-Normwert für den jeweiligen Patienten. Die Narkoseeinleitung erfolgt über einen intravenösen Zugang, der im Narkoseeinleitungsraum gelegt wird. Um die gelegentlich auftretenden Injektionsschmerzen von Propofol zu vermeiden, wird die intravenöse Verweilkanüle körperstammnah (Ellenbeuge, Oberarm) gelegt. Dies führt zu einer deutlichen Reduktion der Inzidenz schmerzhafter Sensationen bei Injektion. Zur Einleitung erhalten die Patienten in allen Gruppen eine Bolus-Injektion Propofol (2 mg/kg). Dieses wird in jeder Gruppe über einen bestimmten Zeitraum injiziert:

Gruppe 1: 5 s

Gruppe 2: 120 s

Gruppe 3: 240 s.

Bei Aussetzen der Spontanatmung werden die Patienten wie üblich mit reinem Sauerstoff über eine Beatmungsmaske beatmet. Es werden bei jedem Patienten folgende Zeitpunkte notiert:

1. Eintreten der Bewußtlosigkeit (der Zeitpunkt, zu dem der Patient nicht mehr auf Ansprache reagiert)

2. Verlust des Lidreflexes (geprüft ab Bewußtlosigkeit).

Das online ausgewertete EEG wird beobachtet, bis ein Ansteigen des BIS ein Nachlassen der Narkosetiefe anzeigt. Zu diesem Zeitpunkt ist die Untersuchung beendet, es werden in üblicher Weise Propofol und Muskelrelaxans nachinjiziert, der Patient intubiert und die Narkose als Kombinationsverfahren fortgesetzt.

# 3.4 Nebenwirkungen

Das Vorgehen entspricht im gesamten Studienverlauf klinisch üblicher Praxis und beinhaltet keine zusätzliche Gefährdung der Patienten. Die Injektion von Propofol kann in seltenen Fällen zu schmerzhaften Sensationen führen. Die Inzidenz dieser Injektionsschmerzen ist höher bei schneller Injektion. Nahezu vollständig vermeiden läßt sich der Injektionsschmerz bei Verwendung einer ausreichend großen Vene. Aus diesem Grund wird den Patienten die venöse Kanüle in der Ellenbeuge oder am Oberarm gelegt.

4. Flußdiagramm

Untersuchungsablauf:

0 s Zeit

Monitoring:

EEG

EKG Propofol BIS-Anstieg

RR-Messung 2 mg/kg (Untersuchungsende)

Pulsoxymetrie

5. Statistik

In allen drei Gruppen kommen klinisch übliche Narkoseverfahren zum Einsatz. Das zusätzliche Monitoring (EEG) ist nicht invasiv und mit keinem weiterem Risiko für den Patienten behaftet. Es handelt es sich um einen Vergleich üblicher Verfahren. Pro Gruppe werden bis zu 33 Patienten untersucht.

Die Gruppen werden mittels Kruskal-Wallis Test und ANOVA auf biometrische Unterschiede getestet

Folgende Zielparameter werden in den einzelnen Untergruppen bestimmt und mittels ANOVA (zweiseitig) verglichen:

Hauptzielparameter:

1. BIS-Minimum

Nebenzielparameter:

1. Minimum der Median Frequenz (MF) (EEG Power Spektrum)

2. Minimum der SEF 95 (EEG Power Spektrum)

3. BSR-Minimum (Burst-Suppression-Ratio, Roh-EEG)

4. Zeit bis Bewußtlosigkeit

5. Zeit bis Verlust des Lidreflexes

6. Zeit bis zum Auftreten des BIS-Minimums

7. Zeit bis zum Auftreten des SEF-Minimums

8. Zeit bis zum Auftreten des MF-Minimums

9. Maximale Abweichung der Herzfrequenz vom Ausgangswert

10. Maximale Abweichung des mittleren arteriellen Blutdrucks vom Ausgangswert

Fallzahlplanung:

Die Planung der Fallzahl erfolgte in Zusammenarbeit mit dem Institut für medizinische Statistik und Epidemiologie der Technischen Universität München (Dipl.-Stat. M. Scholz).

Mit einer Fallzahl zwischen 13 und 33 Patienten in jeder der drei Gruppen hat eine einfaktorielle Varianzanalyse 80% Power, auf einem Signifikanzniveau von 5% einen Unterschied in den Mittelwerten zu entdecken, der durch eine Varianz der Mittelwerte, V=Σ(μ-μ)²/3, im Bereich zwischen 20,5 und 55,5 charakterisiert ist. Dabei wird eine gemeinsame Standardabweichung von 14,00 zugrundegelegt. Die angegebenen Bereiche wurden durch Simulationen auf Basis der aus bisherigen Untersuchungen verfügbaren Ausgangswerte (Mittelwert 36, Standardabweichung 14) unter der Bedingung des Mindestnachweises einer Mittelwertsdifferens in Höhe einer Standardabweichung (14,00) ermittelt. Aufgrund der Unsicherheit mit denen die Annahmen behaftet sind, wird nach n=20 Patienten in jeder Gruppe eine verblindete Zwischenauswertung durchgeführt und die Fallzahl ggf. korrigiert, wobei eine Korrektur lediglich nach oben erfolgen kann. Die maximale Fallzahl wird mit 33 Patienten pro Gruppe festgesetzt.

6. Untersucher

Dr. Gerhard Schneider, Assistenzarzt an der Klinik für Anaesthesiologie

Nicole Forster, Assistenzärztin an der Klinik für Anaesthesiologie

Dr. Jan Martin, Assistenzarzt an der Klinik für Anaesthesiologie

Karl Naguib, Assistenzarzt an der Klinik für Anaesthesiologie

Katrin Praeger, Ärztin im Praktikum an der Klinik für Anaesthesiologie

Univ.-Prof. Dr. Christian Werner, Oberarzt an der Klinik für Anaesthesiologie

Univ.-Prof. Dr. Eberhard Kochs, Direktor der Klinik für Anaesthesiologie

Außer den genannten nehmen keine weiteren Personen an der Untersuchung teil.

7. Literaturverzeichnis

1. Dutton RC, Smith WD, Smith NT: Does the EEG predict anesthetic depth better than cardiovascular variables? Anesthesiology 73(3A), 1990

2. Bowdle TA, Ward RJ: Induction of anesthesia with small doses of sufentanil or fentanyl: dose versus EEG response, speed of onset, and thiopental requirement. Anesthesiology 70(1): 26-30, 1989

3. Forrest FC, Tooley MA, Saunders PR, Prys-Roberts C: Propofol infusion and the suppression of consciousness: the EEG and dose requirements. British Journal of Anaesthesia 72(1): 35-41, 1994

4. Sebel PS, Lang E, Rampil IJ, White PF, Cork R, Jopling M, Smith NT, Glass PS, Manberg P: A multicenter study of bispectral electroencephalogram analysis for monitoring anesthetic effect. Anesthesia & Analgesia 84(4): 891-9, 1997

5. Kearse L, Rosow C, Connors P, Denman W, Dershwitz M: Propofol Sedation/Hypnosis and Bispectral EEG Analysis in Volunteers. Anesthesiology 83(3A): A 506, 1995

6. Kearse L, Rosow C, Sebel P, Bloom M, Glass P, Howell S, Greenwald S: The bispectral index correlates with sedation/hypnosis and recall: comparison using multiple agents. Anesthesiology 83(3A): A 507, 1995

7. Peacock JE, Spiers SP, McLauchlan GA, Edmondson WC; Berthoud M, Reilly CS: Infusion of propofol to identify smallest effective doses for induction of anaesthesia in young and elderly patients. British Journal of Anaesthesia 69(4): 363-7, 1992

8. Ludbrook GL, Upton RN, Grant C, Gray EC: Brain and blood concentrations of propofol after rapid intravenous injection in sheep, and their relationships to cerebral effects. Anaesthesia & Intensive Care 24(4): 445-52, 1996

8. Patientenaufklärung

s. nächste Seite

**KLINIK FÜR ANAESTHESIOLOGIE**

**KLINIKUM RECHTS DER ISAR,**

**TECHNISCHE UNIVERSITÄT MÜNCHEN**

**(Direktor: Univ.-Prof. Dr. med. E. Kochs)**

**PATIENTENINFORMATION**

Sehr geehrte Patientin, sehr geehrter Patient,

Ihr behandelnder Narkosearzt bittet Sie hiermit, an der Untersuchung über den ”Einfluß unterschiedlicher Injektionsgeschwindigkeiten auf den EEG-Effekt von Propofol” teilzunehmen. Im folgenden soll der Hintergrund dieser Untersuchung kurz erläutert werden.

Bei dem für Sie vorgesehenen Einschlafmittel (Hypnotikum) ist aufgefallen, daß die Geschwindigkeit der Gabe die Tiefe des resultierenden Schlafes beeinflusst. Der optimale Zeitraum für die Gabe des Hypnotikums ist jedoch hierbei nicht bekannt. In der vorliegenden Untersuchung verabreichen wir das Medikament Propofol mit einer von drei vorher bestimmten Geschwindigkeiten und beobachten dann, wie schnell Sie einschlafen und wie tief Ihr Schlaf ist.

Die Überwachung der Narkosetiefe geschieht dabei durch die Messung der Hirnströme (EEG). Diese messen wir über Klebeelektroden (ähnlich dem EKG), die wir an Ihrer Stirn anbringen. Aus dem EEG wird dann der Bispektral-Index (BIS) errechnet, der Rückschlüsse auf die Narkosetiefe erlaubt.

Diese Untersuchung ist für Sie nicht belastend und gefährdet Sie nicht zusätzlich. Da Sie für den geplanten operativen Eingriff eine sog. Intubationsnarkose (Vollnarkose) benötigen, unterscheidet sich Ihre Narkose nur in einem Punkt von dem sonst üblichen Vorgehen: die Geschwindigkeit, mit der Ihnen das Einschlafmittel verabreicht wird, ist von vorne herein durch eine zufällige Zuordnung bestimmt. Die Menge des verwendeten Einschlafmittels (Hypnotikums) unterscheidet sich nicht von der üblich verwendeten Dosis. Die verschiedenen Geschwindigkeiten, mit der wir diese Menge verabreichen, sind ebenfalls die gleichen, die wir jeden Tag benutzen. Die Besonderheit hierbei ist lediglich, daß bei Ihnen im voraus feststeht, mit welcher Geschwindigkeit dies bei Ihnen passieren wird.

Sie können jederzeit ohne Angabe von Gründen aus der Untersuchung ausscheiden, ohne daß Ihnen daraus irgendein Nachteil entsteht.

Zur Beantwortung weiterer Fragen stehen wir Ihnen gerne zur Verfügung.

Vielen Dank für Ihre Mitarbeit.

**KLINIK FÜR ANAESTHESIOLOGIE**

**KLINIKUM RECHTS DER ISAR,**

**TECHNISCHE UNIVERSITÄT MÜNCHEN**

**(Direktor: Univ.-Prof. Dr. med. E. Kochs)**

**EINWILLIGUNGSERKLÄRUNG**

Name: __________________ Vorname: __________________ Geburtsdatum: ____________

Hiermit erkläre ich mich einverstanden, an der Untersuchung ”Der Einfluß unterschiedlicher Injektionsgeschwindigkeiten auf den EEG-Effekt von Propofol” teilzunehmen. Diese Untersuchung bestimmt die Auswirkungen der Geschwindigkeit, mit der das Einschlafmittel (Hypnotikum) Propofol gespritzt wird, auf mein Einschlafen zur Narkose.

Ich bin über Ziel und Inhalt der Untersuchung, die praktische Durchführung sowie die damit verbundenen möglichen Risiken, die den allgemeinen Risiken einer Vollnarkose entsprechen, ausführlich aufgeklärt worden. Ich hatte genügend Zeit, meine Entscheidung zu treffen sowie ich die Art und den Zweck der Untersuchung verstehe.

Ich bin berechtigt, jederzeit ohne Angabe von Gründen aus der Untersuchung auszuscheiden. Wenn ich mich dazu entschließe, wird diese Entscheidung in keiner Weise die Qualität meiner Behandlung oder meiner ärztlichen Betreuung beeinflussen.

Mit der Weiterleitung von anonymisierten Daten und Erkenntnissen, die sich aus der Studie ergeben, bin ich einverstanden. Ein Exemplar der Aufklärung und Einverständniserklärung habe ich erhalten.

Ich habe keine weiteren Fragen.

Ich gebe freiwillig die Zustimmung zur Teilnahme an o.g. Untersuchung.

Ort und Datum: _____________________________

_____________________________ ______________________________

Unterschrift des Patienten Unterschrift des Arztes
